# Differential expression of GLP1R, PCSK9, and SGLT2 in human aortic versus mitral valve tissues: exploratory findings in calcific aortic valve disease

**DOI:** 10.3389/fcvm.2026.1813717

**Published:** 2026-04-29

**Authors:** Shou-Quan Cheng, Shuo Fu, Nai-Feng Liu

**Affiliations:** 1 Department of Cardiology, Affiliated Hospital of Xuzhou Medical University, Xuzhou, Jiangsu, China; 2 Department of Cardiology, Zhongda Hospital, School of Medicine, Southeast University, Nanjing, Jiangsu, China

**Keywords:** calcific aortic valve disease, GLP1R, immunohistochemistry, PCSK9, SGLT2

## Abstract

**Background:**

Calcific aortic valve disease (CAVD) is a common valvular heart disorder for which effective pharmacological treatments remain lacking. This study aimed to compare the expression and potential pathophysiological relevance of glucagon-like peptide-1 receptor (GLP1R), proprotein convertase subtilisin/kexin type 9 (PCSK9), and sodium-glucose cotransporter 2 (SGLT2) in human aortic valve (AV) and mitral valve (MV) tissues from patients undergoing valve replacement surgery.

**Methods:**

Surgically resected AV (*n* = 10) and MV (*n* = 13) tissue specimens were collected between March 2020 and December 2022. Target protein expression was assessed via immunohistochemistry (IHC), and valvular calcification was confirmed by Von Kossa staining. Subgroup analyses were performed within calcified vs. non-calcified AV tissues and within a bicuspid aortic valve (BAV) subset (*n* = 3 per group).

**Results:**

GLP1R, PCSK9, and SGLT2 expression levels were significantly elevated in AV tissues compared to MV tissues (all *P* < 0.001). Within calcified AV specimens, expression trends were higher than in non-calcified AV tissues, though these differences did not reach statistical difference (all *P* > 0.05). In the exploratory BAV subgroup (*n* = 3 per group), expression of all three targets was higher in calcified vs. non-calcified tissues (all *P* < 0.05).

**Conclusion:**

These findings reflect differential expression between anatomically distinct valve types and do not establish CAVD-specific upregulation.

## Introduction

Calcific aortic valve disease (CAVD) is the most prevalent valvular heart disease in developed countries, and aortic stenosis (AS) is currently the third most common cardiovascular disorder after coronary artery disease and hypertension. With progressive population aging, the global healthcare burden associated with CAVD continues to rise. Once considered a passive degenerative process, CAVD is now recognized as an actively regulated pathological condition characterized by chronic inflammation, lipid infiltration, oxidative stress, extracellular matrix remodelling, and osteogenic differentiation of valvular interstitial cells (VICs) (1). Despite advances in understanding its pathophysiology, aortic valve replacement remains the only effective treatment, as no pharmacological therapy yet has been proven to halt or reverse disease progression.

In recent years, the concept of cardiometabolic disease (CMD) has gained increasing recognition, highlighting the interplay between metabolic dysregulation and cardiovascular structural damage. Several metabolic therapeutic agents—including glucagon-like peptide-1 receptor (GLP1R), proprotein convertase subtilisin/kexin type 9 (PCSK9), and sodium-glucose cotransporter 2 (SGLT2) have indicated substantial cardiovascular benefits beyond glycemic or lipid control. Experimental studies indicate that GLP-1 receptor agonists (GLP-1RAs) inhibit vascular calcification via activation of the PI3K/Akt/mTOR pathway (2); PCSK9 inhibitors reduce coronary artery calcification progression beyond statin therapy (3); and SGLT2 inhibitors attenuate vascular calcification by suppressing NLRP3 inflammasome activation and endoplasmic reticulum stress (4). Furthermore, observational evidence suggests that SGLT2 inhibitors may delay the progression of AS (HR = 0.61) (5).

However, the expression patterns and potential involvement of these metabolic regulatory targets within human CAVD valvular tissues remain largely unexplored. Therefore, this study aimed to evaluate the expression of GLP1R, PCSK9, and SGLT2 in human aortic valve tissues using immunohistochemistry and to explore their potential association with vascular calcification.

## Methods

### Study population

Patients who underwent surgical valvular resection at the Department of Cardiac and Great Vascular Surgery, Affiliated Hospital of Xuzhou Medical University, between March 2020 and December 2022 were consecutively enrolled. The indications for surgery were severe valvular dysfunction requiring valve replacement according to current clinical guidelines, including severe aortic stenosis or severe mitral valve disease. Inclusion criteria were: age >18 years and availability of complete preoperative transthoracic echocardiography and computed tomography angiography (CTA) data. Exclusion criteria included statins use within 1 month prior to surgery; history of myocardial infarction; active infection; moderate to severe renal insufficiency or chronic liver disease; alcoholism or substance abuse; hypothyroidism or parathyroid dysfunction; active connective tissue disease; recent bleeding events; long-term use of corticosteroids or non-steroidal anti-inflammatory drugs; and Marfan syndrome. Finally, 10 aortic valve (AV) specimens and 13 mitral valve (MV) specimens were included in the final analysis.

### Ethical statement

This study was approved by the Ethics committee of Clinical Investigation of Affiliated Hospital of Xuzhou Medical University on December 10, 2019 (XYFY2019-JS001-02). With prospective ethical approval obtained prior to the commencement of patient enrollment. Written informed consent was obtained from all participants prior to enrollment. Demographic and cardiovascular risk factor data were collected from the hospital's electronic record system.

### Echocardiography

Echocardiographic examinations were performed by experienced sonographers using GE Vivid E95 or Philips EPIQ 7C ultrasound systems. All measurements were obtained in accordance with the standardized guidelines for echocardiographic evaluation of adult valvular heart disease in China (6). Preoperative transthoracic echocardiography was completed for all patients, with assessments of valvular structure, function, and hemodynamic parameters in line with the above guidelines.

### Computed tomography examination

Cardiac CT examinations were performed using a Siemens dual-source CT scanner. Prospective electrocardiogram-gated triggering was applied for calcification assessment. Scanning parameters included a 2 mm × 32 mm × 0.75 mm collimation, 330 ms rotation time, tube voltage of 100–120 kV, and a tube current of 320 mA. Images were reconstructed with a slice thickness of 0.75 mm and a reconstruction interval of 0.5 mm on a dedicated post-processing workstation. All scans were independently interpreted by two experienced radiologists blinded to clinical data. Aortic valve calcification was defined as high-density lesions (130 Hounsfield Units) involving the aortic valve leaflets or aortic root, present in at least three consecutive pixels. Patients with positive findings on both echocardiography and CT were classified into the calcification group. CT imaging was also used to assess for evidence of systemic vascular disease in all patients, with images reviewed for coronary artery calcification, aortic wall changes, and peripheral vascular abnormalities.

### Immunohistochemistry (IHC)

Paraffin-embedded valve tissue sections (4 μm thick) were subjected to immunohistochemical staining for GLP1R, PCSK9, and SGLT2. After deparaffinization and rehydration, the sections were subjected to heat-induced antigen retrieval using citrate buffer (pH: 6.0) in a pressure cooker, maintaining boiling for 2 min. After naturally cooling to room temperature, the sections were rinsed with PBS buffer (0.01 M, pH: 7.4) three times for 5 min each. Endogenous peroxidase activity was blocked with 3% hydrogen peroxide for 10 min at room temperature, followed by blocking with 5% bovine serum albumin (BSA) for 30 min to reduce non-specific binding. Sections were incubated with primary antibodies overnight at 4 °C, followed by incubation with appropriate secondary antibodies (rabbit anti-mouse/rabbit IgG, 1:10,000 dilution in 5% BSA) for 30 min at room temperature. Primary antibody details: GLP1R (Proteintech, Catalog No. 20782-1-AP, China, Dilution 1:1,500, RRID: AB_2884196); PCSK9 (Proteintech, Catalog No. 14425-1-AP, China, Dilution 1:1,500, RRID: AB_2884201); SGLT2 (Proteintech, Catalog No. 24654-1-AP, China, Dilution 1:1,500, RRID: AB_2750601). Immunoreactivity was visualized using 3,3′-diaminobenzidine (DAB), and nuclei were counterstained with hematoxylin. Positive expression was identified as brownish-yellow cytoplasmic staining with blue nuclear counterstaining.

Images were observed and captured using an Olympus BX53 microscope at 200× and 400× magnification with a DP80 digital camera and cellSens software. Five non-overlapping fields were randomly selected for each section. Semi-quantitative analysis was performed using Image-Pro Plus 6.0 software. The integrated optical density of positive staining in each field was measured and divided by the area of the corresponding region to obtain the mean optical density value, which represents the relative expression level of the protein. All photomicrographs are presented in their original form, with only uniform adjustments to brightness and contrast; no modifications were made to the image content.

The scoring of all immunohistochemical sections was independently completed by two pathologists who were blinded to the clinical data. Thirty percent of the sections were randomly selected and re-scored by the same pathologist after a two-week interval to assess intra-observer consistency. The inter-observer and intra-observer intraclass correlation coefficients were both greater than 0.85, indicating good reproducibility of the scoring results.

Positive and negative controls were established for all IHC experiments: human pancreatic tissue (GLP1R), human liver tissue (PCSK9), and human kidney tissue (SGLT2) were used as positive controls; incubation with PBS buffer instead of the primary antibody served as the negative control to ensure staining specificity.

### Von Kossa staining

This was performed to detect calcium salt deposits, which appear black or dark brown. In short, paraffin sections were prepared and treated with silver nitrate, followed by exposure to UV light; calcium salts from the tissue reacted with silver ions and reduced silver to metallic silver, resulting in black or dark brown stain. Prior to counterstaining for contrast between tissues, sodium thiosulphate eliminated any remaining silver ions in the tissue. Thus, all the sections with evidence of calcification were black or brown, showing mineralization; however, the sections without evidence of calcification showed little to no positive staining. Therefore, the results from this technique would support and confirm the findings on the calcification process obtained with immunohistochemistry. Stained sections were observed at 100× magnification, and a scale bar (500 μm) was added to all pathological images for spatial reference.

### Statistical methods

Statistical analyses were performed using SPSS software (version 26.0). Continuous variables were expressed as mean ± standard deviation or median (interquartile range), depending on data distribution, and compared using Student's *t*-test or the Mann–Whitney *U* test, as appropriate. Categorical variables are presented as frequencies (percentages) and analyzed using Fisher's exact test. A two-tailed *P*-value < 0.05 was considered statistically significant. Mitral valve tissue was primarily used in this study as a technical control for surgical handling and tissue fixation. Although some mitral valve specimens (3/13, 23%) also exhibited calcification, the small sample size of the calcified subgroup (*n* = 3) precluded statistically meaningful stratified subgroup analysis. To verify whether the calcified specimens in the mitral valve group influenced the main comparison between aortic and mitral valves, we performed a sensitivity analysis, excluding the calcified mitral valve specimens and re-comparing the expression differences between non-calcified mitral valves and aortic valves.

## Results

### Study population

No statistically significant differences were observed in baseline demographic or clinical characteristics between the AV and MV groups, including age, sex, body mass index (BMI), smoking status, alcohol consumption, hypertension, or diabetes mellitus (all *P* > 0.05) (Table 1). Exploratory analysis within the entire aortic valve group (*n* = 10) comparing hypertensive (*n* = 4) and non-hypertensive (*n* = 6) patients showed no statistically significant differences in the expression of GLP1R, PCSK9, or SGLT2 (all *P* > 0.05). A sensitivity analysis excluding the 3 calcified MV specimens confirmed that GLP1R, PCSK9, and SGLT2 expression remained significantly higher in AV tissues compared to non-calcified MV tissues (all *P* < 0.001), with effect sizes consistent with the original analysis.

**Table 1:** Characteristics of study participants.

Clinical features	Mitral valve (*n* = 13)	Aortic valve (*n* = 10)	*t*/*Z*	*P*
Sex			—	1.000
Male	7 (53.8)	5 (50.0)		
Female	6 (46.2)	5 (50.0)		
Age (y)	58.85 ± 8.41	57.7 ± 8.08	0.329	0.745
Height (cm)	162.31 ± 8.11	162.7 ± 8.18	0.115	0.910
Weight (kg)	65.69 ± 13.15	61.8 ± 10.79	0.759	0.456
BMI (kg/m^2^)	24.75 ± 3.64	23.28 ± 3.29	0.999	0.329
Smoking	3 (23.1)	1 (10.0)	—	0.604
Drinking alcohol	2 (15.4)	2 (20.0)	—	1.000
Hypertension	3 (23.1)	4 (40.0)	—	0.650
Diabetes mellitus	1 (7.7)	1 (10.0)	—	1.000
Statin	1 (7.7)	1 (10.0)	—	1.000
Cerebral infarction	2 (15.4)	0 (0.0)	—	0.486
Cr (umol/L)	66.31 ± 17.46	60.6 ± 17.24	0.781	0.443
Urea (umol/L)	6.35 ± 1.59	5.98 ± 1.33	0.601	0.554
Cystatin c (mg/L)	0.97 (0.89,1.02)	0.91 (0.80,0.98)	−1.585	0.113
Ca^2+^ (mmol/L)	2.26 ± 0.14	2.24 ± 0.11	0.432	0.670
Calcification			—	0.221
N	10 (76.9)	5 (50.0)		
Y	3(23.1)	5(50.0)		

BMI—body mass index; Cr—creatinine; Ca^2+^—calcium.

### Comparison of target expression between aortic and mitral valve tissues

**Figure 1:**
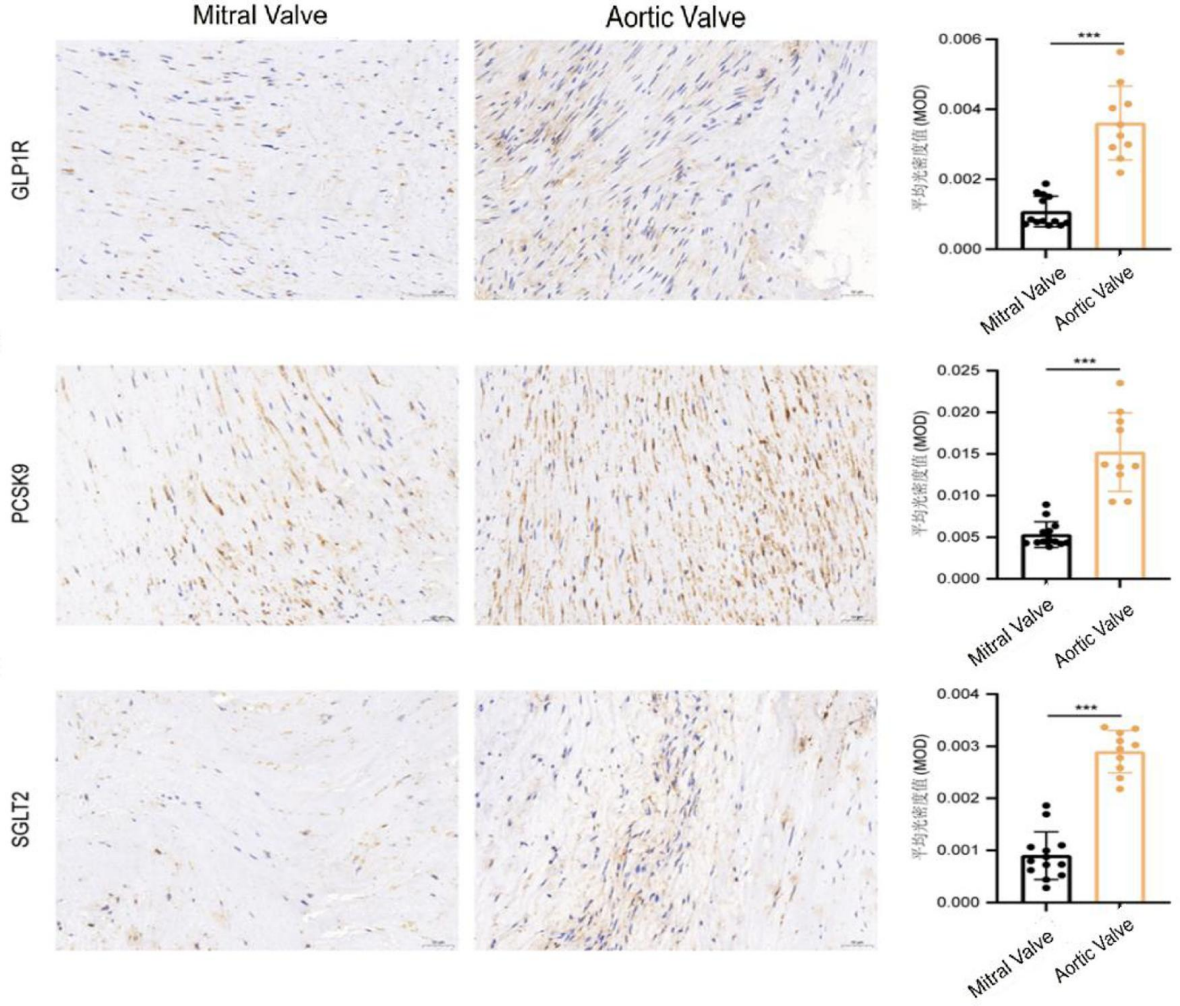
Comparison of target expression between aortic and mitral valve tissues. (All axis titles, scale labels, legend descriptions are in standard English).

Immunohistochemical analysis results indicated significantly higher expression levels of GLP1R, PCSK9, and SGLT2 in AV tissues compared with MV tissues (all *P* < 0.001) (Table 2; Figure 1), indicating enhanced metabolic target activation in aortic valve pathology.

**Table 2:** Comparison of target expression between aortic and mitral valve tissues.

Group	Number	GLP1R	PCSK9	SGLT2
MV	13	0.0011 ± 0.0004	0.0053 ± 0.0015	0.0009 ± 0.0005
AV	10	0.0036 ± 0.0011	0.0152 ± 0.0047	0.0029 ± 0.0004
t	—	7.129	6.396	10.860
*P*	—	<0.001	<0.001	<0.001

After excluding 3 calcified MV specimens, the expression levels of GLP1R, PCSK9 and SGLT2 in non-calcified MV tissues (*n* = 10) were 0.0010 ± 0.0003, 0.0051 ± 0.0014 and 0.0008 ± 0.0004, respectively, and the expression in AV tissues (*n* = 10) was still significantly higher than that in non-calcified MV tissues (all *P* < 0.001).

### Comparison of target expression between calcified and non-calcified tissues

**Figure 2:**
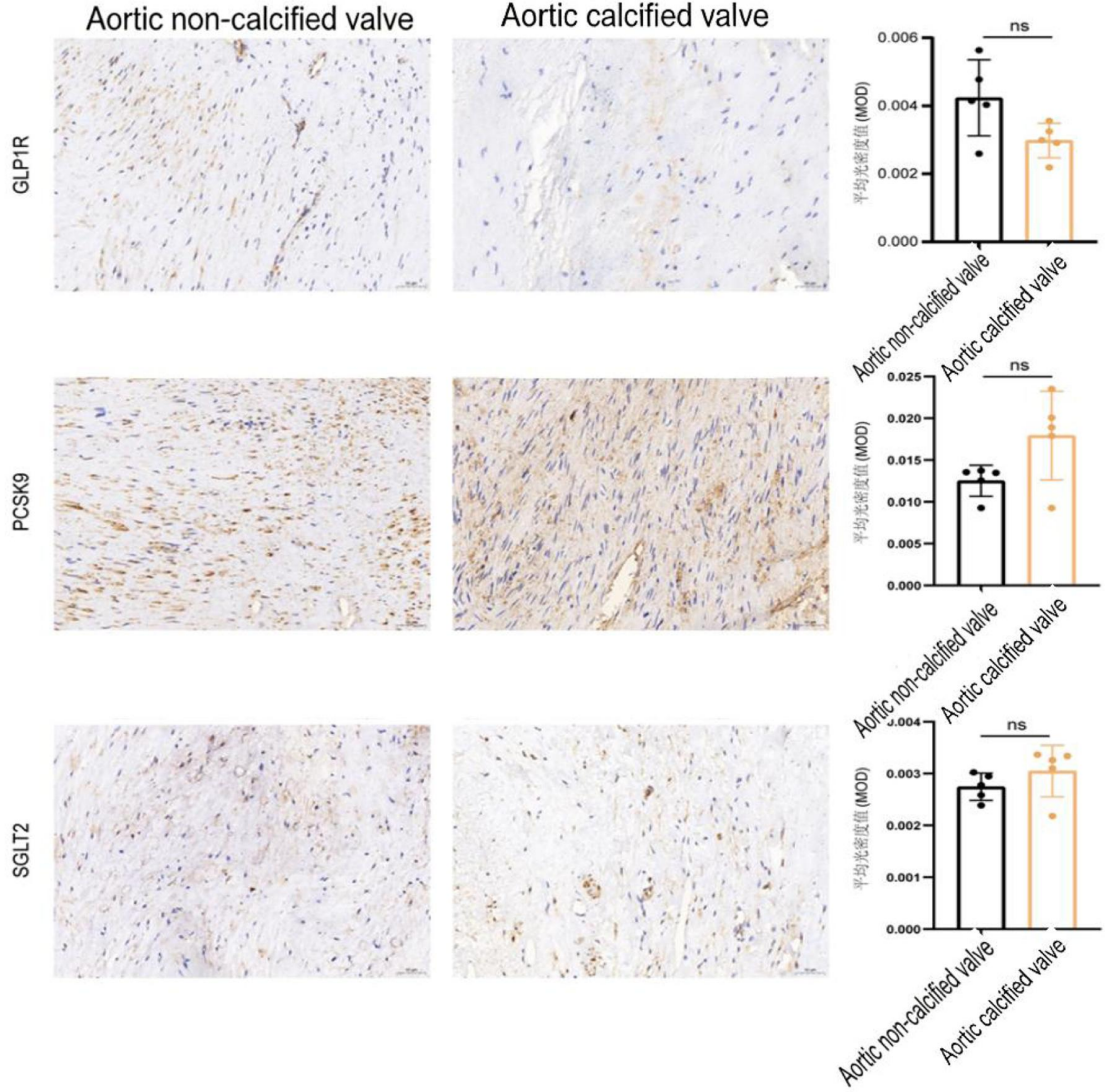
Comparison of target expression between calcified and Non-calcified tissues. (All axis titles, scale labels, legend descriptions are in standard English).

Within the AV group, GLP1R, PCSK9, and SGLT2 expression levels showed a higher expression trend in the calcified tissues compared with non-calcified tissues; however, these differences did not reach statistical significance (*P* > 0.05) (Table 3; Figure 2), possible due to limited sample size. All findings of this study are preliminary observational results. Constrained by the small sample size, all statistical test results are only for trend reference and do not represent statistically validated definitive facts. Further validation is required in larger, adequately powered cohort studies. Notably, the “non-calcified” AV specimens were obtained from patients with aortic valve disease requiring surgery and likely exhibited early pathological changes (e.g., fibrosis, inflammation), rather than representing healthy aortic valve tissue.

**Table 3:** Comparison of target expression between calcified and Non-calcified tissues.

Group	Number	GLP1R	PCSK9	SGLT2
Non-calcification	5	0.0030 ± 0.0005	0.0125 ± 0.0019	0.0027 ± 0.0003
Calcification	5	0.0042 ± 0.0011	0.0179 ± 0.0053	0.0030 ± 0.0005
t	—	2.287	2.156	1.213
P	—	0.052	0.063	0.260

**Figure 3:**
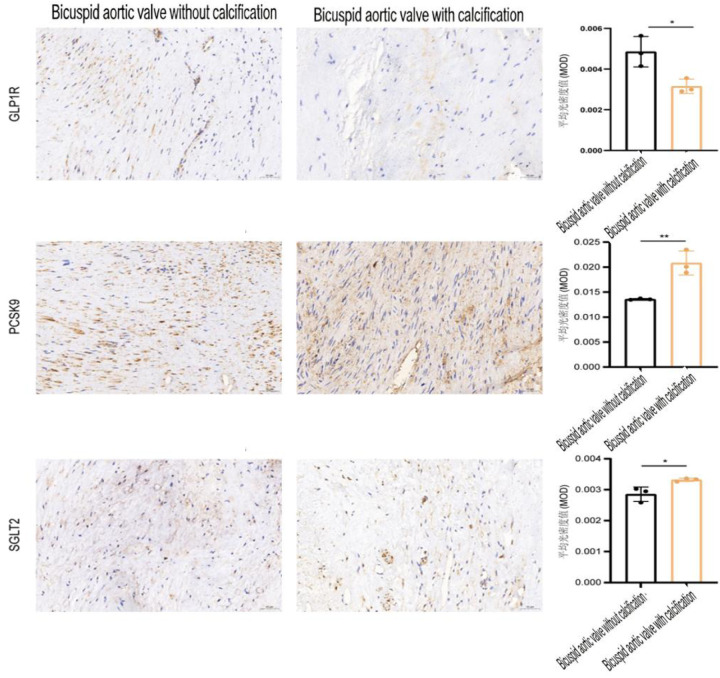
Immunohistochemical expression between the aortic bicuspid valve malformation calcification group and the non-calcification group.descriptive statistics only, no formal statistical tests performed. (All axis titles, scale labels, legend descriptions are in standard English; no significance asterisks are presented).

Of note, within the bicuspid aortic valve (BAV) subgroup (3 calcified, 3 non-calcified), the expression of all three targets exhibited elevated expression levels in the calcified tissues compared with the non-calcified tissues (all *P <* 0.05) (Table 4; Figure 3). These findings are strictly exploratory and hypothesis-generating due to the extremely small sample size in this subgroup, and no definitive mechanistic conclusions should be drawn from this analysis. All statistical test results of this subgroup are only for trend reference and do not represent statistically validated definitive facts, and no statistical inferences are made for this subgroup analysis.

**Table 4:** Immunohistochemical expression between the aortic bicuspid valve malformation calcification group and the non-calcification group. Descriptive statistics only, no formal statistical tests performed.(Exploratory analysis, *n*=3 per group).

Group	Number	GLP1R	PCSK9	SGLT2
Non-calcification	3	0.0032 ± 0.0004	0.0136 ± 0.0001	0.0029 ± 0.0002
BAV calcification group	3	0.0049 ± 0.0007	0.0208 ± 0.0024	0.0033 ± 0.0001

Interestingly, the prevalence of hypertension showed a lower trend in the calcified subgroup (*P* < 0.05), a finding that may reflect the small sample size and warrants cautious interpretation. Given the small sample size of the BAV subgroup, multivariate regression analysis could not be performed to adjust for hypertension as a potential confounding factor. All findings of this study are preliminary observational results, pending validation in a larger and adequately powered cohort.

### Von Kossa staining results

**Figure 4:**
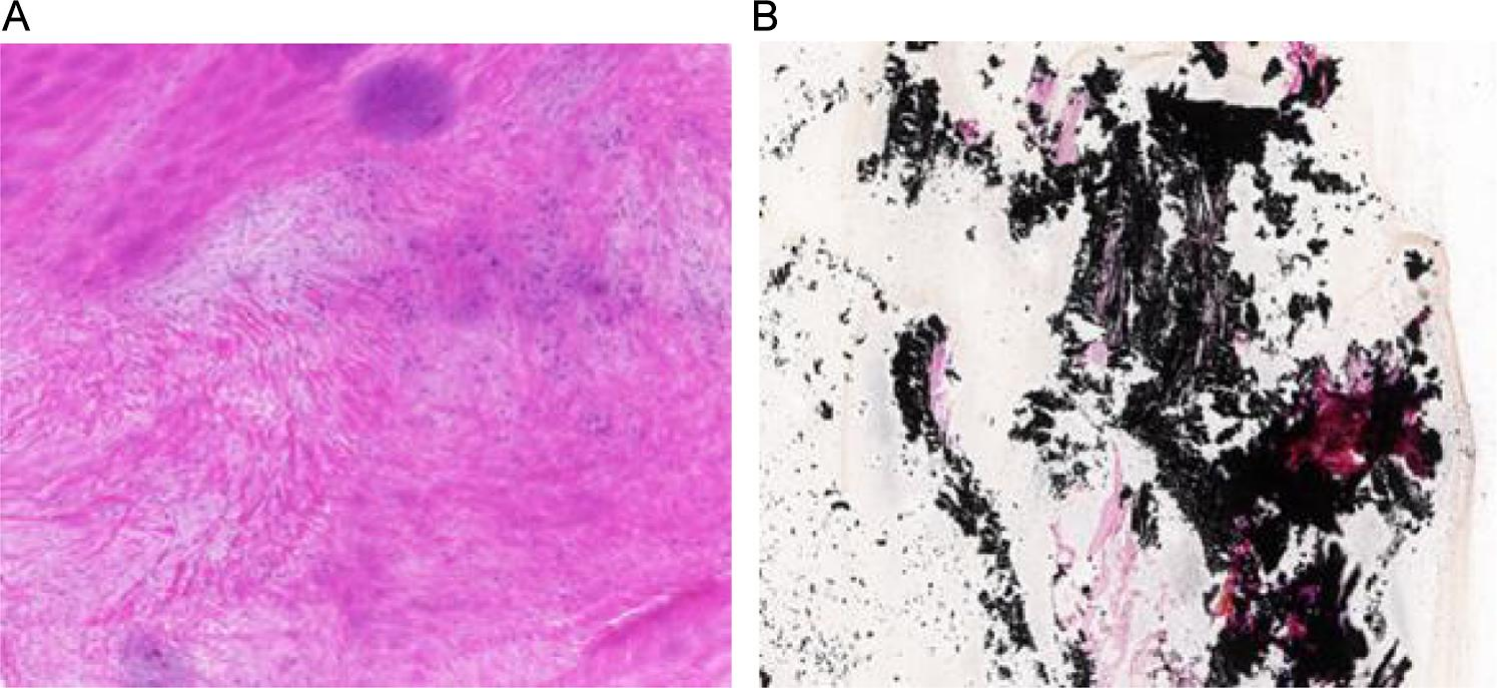
Von kossa staining comparison of valve tissues. (100× magnification, 500 μm scale bar; **(A)** non-calcified valve tissue with normal histological architecture and no Von Kossa-positive staining; **(B)** calcified valve tissue with thickened leaflets, disorganized cell arrangement, and abundant blackish-brown calcium salt deposits (Von Kossa-positive).).

This staining was used to confirm the presence of calcium deposits in the calcified group of patients (Figure 4) through examination of the pathological structure; while significant structural alterations of the valvular tissues were observed due to changes in the normal architecture of the valve and the disorganization of cells, there was an accumulation of large black-dotted or dark brown within the extracellular matrix that would indicate significant accumulation of calcium salts in the calcified tissues. In contrast, the architecture of the non-calcium subgroup of valvular tissue appeared to be preserved; with an orderly arrangement of cells and no evidence of visible mineralization.

### Cellular localization of GLP1R, PCSK9, and SGLT2 in valvular tissues

Immunohistochemical morphological analysis combined with cell marker auxiliary identification revealed the specific cellular localization of each target protein in valvular tissue:

GLP1R: Positive staining was primarily localized in the cytoplasm of valvular interstitial cells (VICs), with prominent staining in VICs surrounding calcific nodules. Weak to moderate positive signals were also observed in the valve surface endothelial cell layer, and occasional staining in infiltrating inflammatory cells (macrophages/lymphocytes).

PCSK9: Positive staining was widely distributed in VICs, with stronger expression in the fibrosa and spongiosa layers. Osteoblast-like VICs near calcified areas showed robust positive staining; endothelial cells and smooth muscle cells at the valve base also exhibited varying degrees of positive signals.

SGLT2: Positive staining was mainly localized in VICs and microvascular endothelial cells, with significantly enhanced expression in VICs surrounding calcific nodules in calcified tissues. Positive signals were also detected in some infiltrating foam-like macrophages.

## Discussion

Our research has provided new preliminary evidence at the tissue level for increased expression of the GLP1R, PCSK9, and SGLT2 by tissue type and in response to calcific aortic valve disease (CAVD). This up-regulation is most robust in aortic valves compared to mitral valves and is even more pronounced in those with calcific degeneration of the aortic valves associated with bicuspid aortic valves (BAVs); the BAV subgroup findings are exploratory and hypothesis-generating due to the extremely small sample size (*n* = 3 per group), and larger studies are required to validate these observations. All findings of this study are preliminary observational results. Constrained by the small sample size, all statistical test results are only for trend reference and do not represent statistically validated definitive facts. Further validation is required in larger, adequately powered cohort studies. These findings suggest that although metabolic regulatory pathways may exert systemic effects, they may also play localized roles in the pathogenesis of valvular tissue during the development of calcification (7). By demonstrating the presence of elevated levels of these cardiometabolic targets in diseased valvular tissue, our study contributes to new mechanistic understandings of the relationships between metabolic dysregulation and structural valve degeneration.

CAVD is thought to be an active, cell-mediated process that has similarities to atherosclerosis, such as lipid infiltration, chronic inflammation, extracellular matrix remodeling, and osteogenic differentiation of valvular interstitial cells (8). The significantly higher expression of GLP1R, PCSK9, and SGLT2 in aortic compared to mitral valves may be explained by the unique hemodynamic conditions and metabolic microenvironments in which the aortic valve exists. The aortic valve is constantly exposed to high-pressure systemic circulation which provides a greater opportunity for inflammatory signals, oxidative stress, and metabolic activation than what the mitral valve is exposed to (2). The analysis of intrinsic biological differences (anatomical and hemodynamic) between the aortic valve and mitral valve is further strengthened here. A clear distinction is made between valve type-associated expression differences and disease-specific upregulation to avoid overinterpretation of the study results.The differences in expression between the two valve types suggest that metabolic signals may play a role in aortic valve susceptibility to calcific degeneration. Notably, a sensitivity analysis excluding calcified mitral valve specimens confirmed that AV expression of these targets remained significantly higher than non-calcified MV expression, indicating that the observed differences primarily reflect intrinsic biological differences between the two valve types rather than calcification status of the MV group (7, 9).

The increased expression of GLP1R in CAVD tissue is interesting. The receptor has an anti-inflammatory, anti-oxidant and anti-calcifying impact in the vascular smooth muscle cells via modulation of the PI3K/Akt/mTOR signalling pathway and inhibition of osteogenesis (4). The increase in GLP1R expression in calcified valves may be a compensatory response to local inflammation and pro-calcifying stimuli, or may instead indicate increased metabolic stress within valve tissue. Although Mendelian randomization studies have not conclusively established a causal link between GLP-1RA and lower risk of developing CAVD (10), population-based analyses may not fully reflect data captured at a local tissue level. Therefore, our results support a biological basis that GLP-1 pathways exist in calcified valves, and further mechanistic and interventional testing is required. This finding is a preliminary observational result and needs to be validated in larger sample cohorts.

Furthermore, the heightened presence of PCSK9 in calcified heart valves correlates with mounting data connecting PCSK9 to heart disease via the formation of calcium deposits. Traditionally, PCSK9 has been recognized for its participation in LDLR destruction and for being a part of lipid metabolism; however, increasingly data indicates that PCSK9 also has pro-inflammatory and pro-osteogenic actions that do not rely solely on lipids. Inflammation related to cytokines can result from the actions of PCSK9, in addition to oxidative stress and the influencing of the cellular differentiation pathways that are important for calcium deposits. Researchers have found that loss-of-function variants of PCSK9 will decrease risk for developing calcific aortic valve disease (CAVD) (11), which supports PCSK9 as having a possible causative function. Our finding of elevated PCSK9 in heart valves implies that a portion of the cardiovascular effects caused by elevated PCSK9 is due to local PCSK9 signal transduction occurring within valvular connective tissue and that it could contribute to calcium deposit formation by causing transformation of valvular interstitial cells (VICs) into osteogenic types and also through the mineralization of extracellular matrix (ECM) components. Thus, PCSK9 has a pathogenic function that is not limited to the systemic regulation of lipids. This finding is a preliminary observational result and needs to be validated in larger sample cohorts.

Another significant finding is the increased SGLT2 expression found in the CAVD valves. It should be noted that the expression of SGLT2 in cardiac tissue has been a subject of controversy. Early single-cell transcriptomic analyses showed minimal SGLT2 expression in healthy hearts. However, a growing number of recent studies indicate that SGLT2 expression can be induced and upregulated in cardiac tissue under pathological conditions such as heart failure, cardiomyopathy, and valvular heart disease (10). The SGLT2 antibody used in this study (Proteintech, 24654-1-AP) is a KD/KO-validated specific antibody that does not cross-react with other SGLT family members such as SGLT1. We also included positive controls (human kidney tissue) and negative controls to ensure staining specificity. Therefore, the SGLT2 expression signals detected in the valvular tissues of CAVD patients in this study possess reliable specificity. SGLT2 inhibitors have been shown to reduce cardiovascular and renal risk factors independent of glycemic control (anti-inflammatory, antioxidant, antifibrotic) and experimental evidence (preliminary) indicates that SGLT2 inhibition will inhibit NLRP3 inflammasome activation, reduce oxidative stress, and modulate autophagy, all of which are basic pathways associated with accelerated CAVD. Current observational data suggest that SGLT2 inhibitors may also slow the progression of AS, therefore suggesting the importance of this pathway. The increased SGLT2 expression seen in both the calcified and the CAVD valves could indicate local sodium-glucose transport mechanisms or associated metabolic stress responses in the diseased valvular tissue that may be contributing to the calcific remodelling process. Nevertheless, the precise function and regulatory mechanisms of SGLT2 in valvular tissue require further elucidation through functional experiments. This finding is a preliminary observational result and needs to be validated in larger sample cohorts.

Importantly, elevated expression levels of all three metabolic targets was observed in calcified tissues within the BAV subgroup; this finding provides preliminary insight into the heterogeneity of CAVD, but the extremely small sample size (*n* = 3 per group) means this analysis is strictly exploratory and cannot be considered definitive evidence of an association between BAV morphology and metabolic pathway activation. BAV is known to confer increased mechanical stress, altered flow dynamics, and earlier onset of valvular calcification compared with tricuspid aortic valves. Therefore, it is possible that congenital valve morphology may predispose these patients to increased inflammatory and metabolic activation resulting in accelerated osteogenic differentiation and mineral deposition, suggesting a potential interaction between metabolic dysregulation and biomechanical factors as a driver for accelerated calcification in patients with BAV-related CAVD. Nevertheless, larger studies with adequately powered cohorts are required to determine whether such associations truly exist.

Notably, we observed a lower trend in the prevalence of hypertension in the calcified subgroup compared to the non-calcified subgroup within the BAV cohort. This is a noteworthy and paradoxical finding. Our analysis suggests this is most likely attributable to chance bias resulting from the extremely small subgroup sample size (*n* = 3 per group). Furthermore, the definition of hypertension relied on medical history records and did not fully account for blood pressure control levels or medication use, which may have introduced information bias. More importantly, as an established risk factor for CAVD, hypertension could potentially influence the expression of GLP1R, PCSK9, and SGLT2 independently. Although our exploratory analysis within the entire aortic valve group did not reveal a significant association between hypertension status and target expression levels, the limited sample size prevented us from effectively adjusting for the potential confounding effect of hypertension in the BAV subgroup through multivariate analysis. Therefore, the observed expression differences in the BAV subgroup should be interpreted with caution, and the possibility that they are partly influenced by the uneven distribution of hypertension cannot be entirely excluded. Future prospective studies with larger sample sizes, incorporating rigorous collection of blood pressure data and medication information, are needed to more precisely delineate the independent contributions of calcification itself vs. comorbidities like hypertension to the expression of these metabolic targets.

The above data support the concept that CAVD is a multifactorial pathological process involving metabolic dysregulation, chronic inflammation, and mechanical stress that collectively drive valvular calcification. The observed upregulation of GLP1R, PCSK9, and SGLT2 suggests that these pathways may represent potential therapeutic targets in CAVD. Due to the cross-sectional design of the present study, causal relationships cannot be established. However, the tissue-level findings provide biological rationale for investigating pharmacological modulation of these pathways. Therefore, future translational studies incorporating molecular analyses, longitudinal clinical data and pharmacological interventions are required to determine whether targeting these metabolic pathways may influence the progression of CAVD. All findings of this study are preliminary observational results, and the above inferences need to be verified in larger and adequately powered cohort studies.

It is also important to address the lack of efficacy of statin therapy in CAVD, a key clinical consideration. Despite the shared lipid-mediated inflammatory pathways between CAVD and atherosclerosis, statins have failed to show consistent benefit in halting or reversing CAVD progression in clinical trials. This may be due to several factors: (1) CAVD is a multifactorial process with mechanical stress and osteogenic differentiation playing dominant roles, beyond just lipid accumulation; (2) statin therapy is often initiated late in the disease course, when valvular remodelling and calcification are already irreversible; (3) the valvular microenvironment has limited drug penetration, reducing the local effect of systemic statin administration; (4) CAVD involves unique pathological pathways (e.g., osteogenic differentiation of VICs) that are not targeted by statin therapy. The identification of GLP1R, PCSK9, and SGLT2 as local targets in CAVD tissue provides new avenues for therapeutic development, distinct from traditional lipid-lowering strategies.

Obtaining human valve tissue for mechanistic studies is inherently challenging because specimens are only available from patients undergoing valve replacement surgery. Consequently, many histological and molecular studies of calcific aortic valve disease rely on relatively small but carefully characterized cohorts of surgical samples. Unfortunately, due to the retrospective nature of the present study, detailed information regarding symptom duration and some additional imaging variables was not consistently available for all patients, which limited further clinical correlation analysis; this limitation is acknowledged and future prospective studies should incorporate comprehensive clinical phenotyping.

Several limitations should be acknowledged. First, the sample size was relatively small, which may have limited statistical power. In particular, the subgroup analysis involving bicuspid aortic valve specimens included only a very small number of samples, and therefore the results should be interpreted as exploratory and hypothesis-generating rather than conclusive. Because human valvular tissue can only be obtained during valve replacement surgery, many histological studies of calcific aortic valve disease rely on relatively small cohorts of surgical specimens. Second, the cross-sectional design precludes causal inference. Third, functional experiments were not performed to elucidate the precise mechanistic roles of these targets. Fourth, mitral valve tissue was used as a technical control rather than an ideal healthy aortic valve control; non-calcified aortic valve tissue from donor hearts or patients with isolated aortic regurgitation would be a more appropriate comparator but is unavailable due to practical and ethical constraints. Fifth, the “non-calcified” AV specimens were not healthy and likely exhibited early pathological changes, which may have narrowed the expression differences between calcified and non-calcified AV groups. Larger prospective studies incorporating molecular and single-cell analyses are warranted to validate and expand upon these findings.

### Cellular localization implications

Immunohistochemical analysis in this study revealed that GLP1R, PCSK9, and SGLT2 are primarily localized in valvular interstitial cells (VICs) within CAVD valve tissues, with significantly enhanced expression particularly in interstitial cells surrounding calcific nodules. VICs are the most abundant cell type in valvular tissue, responsible for maintaining extracellular matrix homeostasis. Under pathological stimulation, they undergo osteoblast-like differentiation and are key cells in the formation of calcific nodules. Therefore, the upregulation of these three metabolic targets in VICs suggests that they may be directly involved in regulating the osteogenic differentiation process of these cells. Furthermore, the expression of SGLT2 in microvascular endothelial cells and the occasional positive signals for PCSK9 in infiltrating inflammatory cells also suggest that these targets may indirectly influence the calcification process by modulating endothelial function and local inflammation. This cellular localization information provides an important morphological basis for understanding the local role of metabolic pathways in CAVD.This finding is a preliminary observational result and needs to be validated in larger sample cohorts.

## Conclusion

This study shows significantly higher expression of GLP1R, PCSK9, and SGLT2 in human aortic valve tissues compared with mitral valve tissues obtained from patients undergoing valve replacement surgery. Whether, this differential expression reflects intrinsic biological differences between the two valve types including their distinct embryologic origins, hemodynamic environments, and baseline transcriptional profiles—or represents disease-associated upregulation related to CAVD pathology cannot be determined from the present data, as healthy control valve tissues were not available for comparison.

Within the aortic valve group, expression trends were higher in calcified vs. non-calcified tissues, though these differences did not reach statistical significance. Exploratory observations in a small bicuspid aortic valve subgroup (*n* = 3 per group) showed elevated expression in calcified specimens, generating hypotheses that warrant further investigation but requiring no definitive conclusions to be drawn. All findings presented are preliminary and descriptive in nature. Constrained by the small sample size and the absence of healthy control tissues, statistical comparisons serve as trend references rather than validated definitive facts. Further validation incorporating healthy valve controls (e.g., from heart transplant donors or autopsy specimens), larger, and adequately powered patient cohorts. comprehensive clinical phenotyping, and functional experiments are necessary to determine whether GLP1R, PCSK9, and SGLT2 are truly upregulated in CAVD pathology and whether these metabolic targets represent viable therapeutic opportunities for delaying disease progression.

## Data Availability

The raw data supporting the conclusions of this article will be made available by the authors, without undue reservation.
